# Algorithms, data structures, and numerics for likelihood-based phylogenetic inference of huge trees

**DOI:** 10.1186/1471-2105-12-470

**Published:** 2011-12-13

**Authors:** Fernando Izquierdo-Carrasco, Stephen A Smith, Alexandros Stamatakis

**Affiliations:** 1 The Exelixis Lab, Scientific Computing Group, Heidelberg Institute for Theoretical Studies, Schloss-Wolfsbrunnenweg 35, D-69118 Heidelberg, Germany; 2 2 Smith Lab, Dept. Ecology and Evolutionary Biology, University of Michigan, 2005 Kraus Natural Science Building, Ann Arbor, MI 48109-1048 USA

## Abstract

**Background:**

The rapid accumulation of molecular sequence data, driven by novel wet-lab sequencing technologies, poses new challenges for large-scale maximum likelihood-based phylogenetic analyses on trees with more than 30,000 taxa and several genes. The three main computational challenges are: numerical stability, the scalability of search algorithms, and the high memory requirements for computing the likelihood.

**Results:**

We introduce methods for solving these three key problems and provide respective proof-of-concept implementations in RAxML. The mechanisms presented here are not RAxML-specific and can thus be applied to any likelihood-based (Bayesian or maximum likelihood) tree inference program. We develop a new search strategy that can reduce the time required for tree inferences by more than 50% while yielding equally good trees (in the statistical sense) for well-chosen starting trees. We present an adaptation of the Subtree Equality Vector technique for phylogenomic datasets with missing data (already available in RAxML v728) that can reduce execution times *and *memory requirements by up to 50%. Finally, we discuss issues pertaining to the numerical stability of the Γ model of rate heterogeneity on very large trees and argue in favor of rate heterogeneity models that use a single rate or rate category for each site to resolve these problems.

**Conclusions:**

We address three major issues pertaining to large scale tree reconstruction under maximum likelihood and propose respective solutions. Respective proof-of-concept/production-level implementations of our ideas are made available as open-source code.

## Background

The rapid accumulation of molecular sequence data that is driven by novel wet-lab sequencing techniques such as pyrosequencing [1] and collaborative whole-genome sequencing projects such as the 10 K vertebrate genome project http://www.genome10k.org/ pose unprecedented challenges with respect to the scalability and numerical stability of phylogenetic inference programs. Datasets are continuously growing with respect to the number of base-pairs and/or the number of taxa. For likelihood-based [2] (Bayesian and Maximum Likelihood) codes with their extremely high computational requirements in terms of memory *and *floating point operations, improving scalability for large datasets is particularly challenging. Here, we focus on algorithm design, improvement of numerical stability, and technical solutions for accelerating the likelihood function and reducing the memory requirements on phylogenomic datasets with missing data that contain more than 10,000 taxa. The concepts we introduce are generic, that is, they can be applied to other likelihood-based programs such as IQPNNI [3], GARLI [4], PHYML 3.0 [5], FastTree 2.0 [6], MrBayes [7], PhyloBayes [8], and BEAST [9] or to libraries for computing the phylogenetic likelihood such as BEAGLE http://code.google.com/p/beagle-lib/.

The largest published maximum likelihood tree to date contained approximately 13,000 taxa [10]. FastTree 2 has been used to infer approximate maximum likelihood trees of approximately 200,000 taxa [6], and the largest published tree using parsimony contained approximately 73,000 taxa [11]. With novel alignment assembly methods such as, for instance, PHLAWD [12], increasing data availability, and collaborative projects for reconstructing huge trees (e.g., the iPlant plant tree of life grand challenge project http://www.iplantcollaborative.org/challenge/iplant-tree-life) there exists a need to infer even larger trees exceeding 100,000 taxa. RAxML version 7.2.8 alpha (available at http://wwwkramer.in.tum.de/exelixis/software.html) already incorporates some of the mechanisms presented here. It has been successfully deployed --without crashing-- to infer biologically reasonable Maximum Likelihood (ML [2]) trees on phylogenomic datasets with 10-20 genes for 38,000, 56,000, and 116,000 taxa.

## Methods

In the following we discuss three different topics: (i) the design of a new search algorithm for large datasets, (ii) an appropriately adapted re-implementation of the Subtree Equality Vector (SEVs [13]) technique in RAxML, and (iii) numerical issues that arise with the widely used Γ model of rate heterogeneity [14].

### PhyNav Revisited: Constraining the tree search to a backbone tree

PhyNav (Phylogenetic Navigator [15]) first introduced the idea to reduce the dimension of the tree (and potentially also the memory footprint of the tree) on which the search is conducted, by identifying subtrees of closely related taxa whose root may be represented by a single *virtual tip*. The rationale is that in large alignments there may exist many taxa that are closely related to each other which can therefore be clustered together into a single virtual tip (which we also denote as super-taxon). By clustering taxa into virtual tips, the dimension of the tree can be reduced allowing for a tree search on the backbone tree that is induced by the virtual tips.

Given a hypothetical perfectly balanced tree, a reduction of 50% could correspond to collapsing each pair of taxa into a single virtual tip. Thus, for each pair of tips, there would be one less inner node to operate on, and the total number of inner nodes would have been halved.

We henceforth denote such a reduction of the tree dimension as *reduction factor *and denote the reduced unrooted tree that is induced by the virtual tips as *backbone *tree.

Once an appropriate backbone tree has been computed (see below), a SPR-based (Subtree Pruning Re-Grafting) search, or any other heuristic search strategy using, for instance, NNI (Nearest Neighbor Interchange) or TBR (Tree Bisection Reconnection) moves, can be restricted to operate within this backbone tree. In other words, the virtual tips are interpreted as tips in the backbone tree on which we conduct the tree search. In our RAxML proof-of-concept implementation that deploys SPR moves, only subtrees that form part of the backbone tree are pruned and will exclusively be re-inserted into branches that lie within the backbone.

Despite restricting the tree search to the backbone, in our setup, we always compute the log likelihood score of the comprehensive tree during the backbone tree search. The log likelihood score of the comprehensive tree can be easily computed, because virtual tips are ancestral probability vectors that summarize the signal of the (excluded) real tips situated below the respective virtual tip. Note that, memory requirements for storing the ancestral probability vector representing a virtual tip are significantly higher than for storing a terminal taxon. For terminal taxa, it suffices to store the molecular sequence as an array of single bytes and to use a lookup table for obtaining the corresponding tip probability vector (see [16] for details).

One way to implement a PhyNav-like method comprises the following computational steps: Initially, generate a reasonable starting tree, using, for instance, parsimony. Then, determine an appropriate backbone tree and optimize branch lengths and model parameters on this comprehensive tree under ML. Thereafter, determine and mark the ancestral probability vectors that will become virtual tips in the backbone. Finally, conduct a tree search on the backbone tree.

To also achieve a memory footprint reduction (not implemented), one can write a multiple sequence alignment for the backbone to file that will partially consist of nucleotide sequences and partially of ancestral probability vectors representing virtual tips. This reduced alignment can then be parsed together with the backbone tree for conducting a tree search. By deploying PhyNav-like algorithms, one can save memory, if inner nodes (ancestral probability vectors) are excluded from the backbone, since ancestral probability vectors largely dominate the memory requirements of likelihood-based programs (see [16] for details).

In terms of algorithm design, the issue that predominantly affects performance is the computation of the backbone tree, that is: How do we determine "good" virtual tips?

#### Building the Backbone

To build a backbone, we assume that a reasonable (i.e., non-random) fully resolved comprehensive tree *T *comprising all taxa (e.g., obtained via parsimony using TNT [11] or RAxML) is provided as input. This comprehensive *n*-taxon tree has *n *tips and *n*-2 ancestral (inner) nodes.

The second parameter for the backbone tree algorithm is the desired tree size reduction factor *R*, where 0.0 <*R *< 1.0. This parameter denotes to which fraction of *n *the backbone tree shall be reduced in size. Ideally, the backbone tree will then comprise *n*·*R*-2 ancestral nodes and *n*·*R *backbone tips. Backbone tips may either be virtual tips (ancestral nodes) or real tips. Evidently, choosing very low values of *R *may significantly impact the quality of the inference, specially if very short branch lengths are present. According to our experience (see results section), using *R *> 0.25 is a safe lower bound.

Our backbone construction algorithm executes two main computational steps that are described in more detail below. Initially, we assign the *n *tips to *n*·*R *clusters, that is, *c *= ⌈*n*·*R*⌉, where *c *is the total number of clusters obtained. For each tip we store a cluster identifier that denotes to which cluster the tip has been assigned. Thereafter, we traverse the tree and use the cluster identifiers to label all ancestral nodes as residing inside, outside or on the boundary of the backbone.

##### Tip clustering

There are plenty of possible approaches to cluster tips. First, the available topology itself (for example, the optimized parsimony starting tree) can be used directly as a hierachical tree (hierachical clustering). Another alternative may be to compute parsimony scores of subtrees, and then cluster together according to a threshold. Here, we only present an approach based on computing a distance matrix and applying average-linkage hierarchical clustering [17]. Our assesment indicated this approach yields significantly better results than the others.

In standard hierarchical clustering, the first step consists of calculating a distance matrix that contains the pair-wise distances between all items (tips) to be clustered. However, given a comprehensive tree *T *with ML estimates of branch lengths, we can directly obtain this distance matrix from the tree by calculating the pair-wise patristic distances. The patristic distance between two taxa is the sum of branch lengths on the path in the tree connecting the two taxa. Thus, the distance matrix is symmetric. The space requirements for storing such a patristic distance matrix are in *O*(*n*^2^) which can become prohibitive for large alignments with *n *≥ 30, 000 tips. We observe that, the pair-wise patristic distances between most tips will be very large and hence these tips will be assigned to different clusters anyway. Therefore, to save memory, one can decompose this process into computing several smaller, partial distance matrices, since the comprehensive starting tree already induces a hierarchical clustering structure. If we subdivide the problem into computing *p *partial pair-wise distance matrices, and each partial matrix defines $c=\sum_{i=0}^{k}c_{i}=\left\lceil n\cdot R\right\rceil$, so that the total number of clusters, we need to ensure that desired clusters still corresponds to the specified reduction factor *R*. To achieve this, we do not fix the number of partial matrices *k *a priori. Instead, we define a threshold value *m *that represents an upper bound for the number of tips contained in each partial matrix. Let *n *be the total number of taxa, *n_i _*the number of tips in a partial matrix, where *n_i _*≤ *m *and $n=\sum_{i=0}^{k}n_{i}$. From each partial matrix, we extract an amount of clusters proportional to its size, that is, $c_{i}\propto c\times \frac{n_{i}}{n}$.

This is implemented as follows: First, we find a set of subtrees such that (i) each subtree has as many tips as possible and at most *m *tree tips and (ii) each tree tip is included in exactly one subtree, that is, all tree tips are included in one subtree and no tip forms part of more than one subtree.

For each such subtree *i*, we then build a (partial) patristic distance matrix for all *n_i _*subtree tips. Thereafter, we cluster them, by generating a hierarchical cluster tree. This hierarchical tree may be cut at different levels to generate a varying number of subtree tip groups. We choose to cut the the tree such that it generates *c_i _*clusters of subtree tips. If required, the number of desired clusters *c_i _*will have been iteratively adjusted beforehand (for further details see below) for each partial matrix *i *to ensure that $c=\sum_{i=0}^{k}c_{i}$.

For example, consider a 40, 000-taxon tree, a reduction factor of 0.5 (corresponding to 20, 000 clusters), and a partial matrix threshold of 32, 000 taxa. In this example, we may obtain distance matrices of 10, 000 and 30, 000 taxa respectively. Then we will need to extract 15, 000 clusters from the 30, 000 taxa distance matrix and 5, 000 clusters from the 10, 000 taxa distance matrix.

To be able to apply this method and compute partial patristic distance matrices, we need to devise an algorithm that selects subtrees from the comprehensive phylogeny such that they contain at most *m *taxa. We start by selecting the innermost node of the tree (see below). Consider that, each inner node *i *of an unrooted binary tree *T *can be regarded as a trifurcation that defines three subtrees *T*_*i, a*_, *T*_*i, b *_and *T*_*i, c*_. We define subtree length *stl*(*T_i_*) as the sum of all branch lengths in subtree *T_i_*. Thus, *stl*(*T*_*i, a*_) + *stl*(*T*_*i, b*_) + *stl*(*T*_*i, c*_) = *stl*(*T*) holds for any inner node *i*, where *T *is the comprehensive tree.

In our current default implementation, we select the innermost node *j *that maximizes *stl*(*T*) - max{*stl*(*T*_*j, a*_), *stl*(*T*_*j, b*_), *stl*(*T*_*j, c*_)}. An alternative criterion for selecting the innermost node is to determine the node that minimizes the variance of the three outgoing subtree lengths. Other possible criteria, that are not based on subtree length may be defined, for instance, as finding the node that minimizes the variance of the node-to-tip distance or finding the node with the highest minimum node-to-tip distance. The node-to-tip distance is defined as the sum of branch lengths on the path in the tree leading from an ancestral node to a tip.

We conducted an empirical assessment (based on our collection of large real-world trees) of these alternative approaches for determining the innermost node of a tree. The outcome (results in additional file 1) was that the respective innermost nodes (as identified by the above criteria) are either identical or close neighbors, that is, located in the same region of the tree.

##### Backbone construction

Once we have determined the innermost node, we conduct a depth-first tree traversal starting at this node and descend into each of the three subtrees. The depth-first traversal terminates, when a subtree root is encountered that comprises ≤ *m *tips. All subtree roots that contain ≤ *m *tips are stored in a list for further processing. Thus, when the depth-first traversal has been completed, this list of *k *subtree roots can be used to generate the *k *partial patristic distance matrices of maximum size *O*(*m*^2^). In our implementation, we set *m *:= 1024. This a suitable value, since only a few seconds are required for processing partial distance matrices.

For each subtree root (i.e., each partial patristic distance matrix), we determine how many clusters should approximately be extracted, via ${\bar{c}}_{i}:=\left\lfloor \frac{1}{2}+c\cdot \frac{n_{i}}{n}\right\rfloor$, where *i *is the cluster (subtree) number, *n_i _*is the number of tips in the respective cluster/subtree, *c *= *n*·*R *is the total number of desired clusters, and ${\bar{c}}_{i}$ is the number of clusters for subtree *i*. In general, $c\neq \sum_{i=0}^{k}{\bar{c}}_{i}$. The overhead, or deficit for that matter, of clusters, that is given by $\Delta c=c-\sum_{i=0}^{k}c_{i}$, is then proportionally distributed across all remaining partial matrices. This process is repeated iteratively until no overhead (or deficit) remains. In each iteration, we reassign $c_{i}:=\left\lceil {\bar{c}}_{i}+\Delta c\cdot \frac{{\bar{c}}_{i}}{c}\right\rceil$ until $c=\sum_{i=0}^{k}c_{i}$ for every *i*.

Then, for each subtree *i *= 1...*k *we proceed as follows:

For all tips in subtree *i*, calculate the patristic distances to all other tips in this subtree and save them in the respective distance matrix.

Apply pairwise average clustering to generate a hierarchical tree of joins from the distance matrix.

Cut the tree, such that exactly *c_i _*clusters are generated.

Add those clusters to a global list of clusters. Maintain a list that keeps track to which cluster a tip belongs.

When all subtrees have been processed, we have a list of *c *clusters. Note that, each cluster contains *x *tips, where 1 ≤ *x *≤ *m *and that each tip is assigned to exactly one cluster. The step to build the backbone from the clusters is not trivial. We use labels (inside, boundary and outside) to identify which nodes belong to the backbone and which ones do not.

The backbone tree is defined by nodes marked as inside and boundary. Once the clusters have been computed, we build the backbone as follows: Initially, we label each inner node in the tree as inside, tip nodes which belong to clusters of size one as boundary, and all remaining terminal nodes as outside. In addition, we maintain a list for storing the cluster identifiers of ancestral nodes that will not form part of the backbone.

Once this is done, we update/adapt the backbone assignment for ancestral nodes: The nodes of the comprehensive tree that represent the *k *subtree roots will remain inside the backbone. On each of the *k *subtree roots, we initiate a post-order traversal to relabel the ancestral nodes, if required, according the following rule set:

If the two child nodes are labeled as inside or boundary, the ancestral node remains labeled as inside.

If one child is labeled as inside or boundary and the other child as outside, the ancestral node is relabeled as inside and the outside child node is relabeled as boundary.

If both children are labeled as outside, we need to check to which cluster they belong. If they belong to the same cluster, the parent node is labeled as outside and the shared cluster identifier of the child nodes is propagated to the parent node. If the two children do not belong to the same cluster, the parent node is labeled as inside and both children are relabeled as boundary.

**Figure 1:**
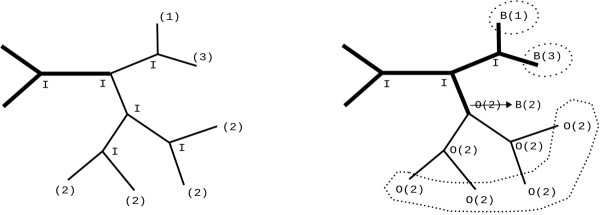
**Consistency of labels at the backbone boundaries**. At first (left) an initial backbone exists (thick branches), all inner nodes are labelled as inside (I) and each tip node has a cluster id. After completion of the post-order traversal (right), each inner node has been relabelled accordingly, if required. Here, cluster 2 is monophyletic, hence the cluster id was inherited propagated back to the initial backbone node. This produced a branch (edge) with an inside and an outside node; therefore the outside(O) node is relabelled (arrow) as boundary(B) node.

When the post-order traversal is about to be completed, we arrive at the subtree root *i *again, which was originally labelled as inside. At this point, we check whether the adjacent backbone node of the subtree root *i *has been labeled as outside. Whenever this is the case (see Figure 1 for an example), the adjacent backbone node is relabeled as boundary for consistency.

**Figure 2:**
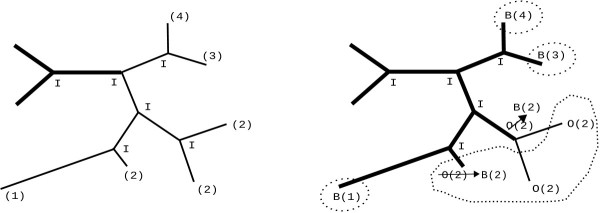
**Increase of in backbone tips due to topology conflicts**. At first (left) an initial backbone exists (thick branches), all inner nodes are labelled as inside (I) and each tip node has a cluster id. Upon completion of the post-order traversal (right), each inner node has been relabelled accordingly. Here, cluster 2 is not monophyletic. Hence, an additional virtual tip is created, that is, cluster 2 generates 2 boundary tips.

Given a set of tips that form part of the same cluster, it may occur that these tips also form a monophyletic group. In this case, during the postorder traversal, all ancestral nodes will be grouped together under the same cluster identifier and the common ancestral node will become a backbone boundary (virtual tip). However, if the tips in a cluster are not monophyletic (see for instance, in Figure 2), the application of the above rules requires some additional boundary relabelling.

Based on the prolegomena, a single cluster may thus induce more than a single virtual tip. As a consequence, the number of virtual tips may actually be higher than the number of clusters. In turn, the reduction of tree size that can be achieved will be smaller than specified by *R*. The impact and frequency of occurrence of this phenomenon (non-monophyletic clusters) depends on the shape of the tree and the branch lengths. In Table 1, we outline this effect for trees with 37,831 and 55,593 taxa. We computed the average number of virtual tips generated by our algorithm on 10 distinct trees per dataset and reduction factors of 0.25 and 0.5 respectively.

**Table 1:** Average number of computed backbone tips over 10 distinct trees

Average number of computed backbone tips		
Reduction Factor R	37831 (expected)	55593 (expected)

0.25	12668.0 (9457.75)	19366.7 (13898.25)

0.50	22340.0 (18915.5)	33501.5 (27796.5)

The average number of backbone tips is higher than the expected number *n*·*R*

#### Tree Searches on the Backbone

We have implemented the above algorithm in a dedicated RAxML version that is available for download at http://wwwkramer.in.tum.de/exelixis/software/BackboneSearch.zip. Initially, RAxML will generate a comprehensive randomized stepwise addition order parsimony tree, or read in a user specified tree via -t. Then it will optimize ML model parameters--including branch lengths--on the comprehensive tree. Thereafter, it will execute the backbone algorithm as described above. The tree searches on the backbone are based on the standard RAxML hill-climbing algorithm. Lazy SPR moves are only conducted within the backbone. After each cycle of SPR moves (see [18] for details), the backbone tree will be re-computed based on the currently best tree. Also, the branch lengths of the entire tree (including those branches not forming part of the backbone) will be re-optimized once after each SPR cycle.

### Subtree Equality Vectors Re-Visited

We introduced and implemented the concept of Subtree Equality Vectors (SEVs) to accelerate likelihood computations by reducing the number of required floating point operations in 2002 [19]. Conceptually similar approaches were presented in 2004 [20] and 2010 [21].

The underlying idea is based on the following observation: Given two identical alignment sites *i *and *j *that evolve under the same evolutionary model (GTR parameters, *α *shape parameter of the Γ function, etc.) and for which a joint branch length has been estimated, their per-site log likelihoods *LnL*(*i*) and *LnL*(*j*) will be identical. Hence, to save computations, one can compress the identical sites into a single site pattern and assign a respective site pattern count (weight) to this site pattern. Thus, for two identical sites *i *and *j*, we can compute the per-site log likelihood as 2·*LnL*(*i*). This global compression of alignments (executed prior to conducting likelihood computations) is implemented in all current likelihood-based codes.

This basic idea of site compression can be extended to the subtree level, by using SEVs for instance, to save additional computations. Consider the equation [2] for computing the ancestral probability vector entry for observing nucleotide A at site *i *of a parent node node *p*, with two child nodes *l *and *r *given the respective branch lengths *b_l _*and *b_r _*and transition probability matrices *P*(*b_l_*) and *P*(*b_r_*):

(1) $${\vec{L}}_{A}^{\left(p\right)}\left(i\right)=\left(\overset{T}{\underset{S=A}{\sum }}P_{AS}\left(b_{l}\right){\vec{L}}_{S}^{\left(l\right)}\left(i\right)\right)\left(\overset{T}{\underset{S=A}{\sum }}P_{AS}\left(b_{r}\right){\vec{L}}_{S}^{\left(r\right)}\left(i\right)\right)$$

We observe that, if the site patterns in the subtree (and hence sub-alignment) rooted at *p *at sites *i *and *j *are identical, *and *if the transition probability matrices *P*(*b_l_*) and *P*(*b_r_*) are identical at sites *i *and *j *(implying identical branch lengths and model parameters at sites *i *and *j*), then $L_{S}^{\left(p\right)}\left(i\right)=L_{S}^{\left(p\right)}\left(j\right)$ for all states *S *(e.g., A, C, G, T). Thus, we can avoid re-computing all ancestral states for site *j *if we have already computed the ancestral states for site *i*.

The key technical challenge with this approach is that it requires a large amount of bookkeeping, to keep track of identical subtree site patterns (for details see [19]). Moreover, SEVs require additional data structures and a case switch in the innermost loop of the likelihood function that iterates over the sites of the ancestral probability vectors, which may lead to cache misses and incorrectly predicted conditional jumps by the processor hardware. Because of these observations we had abandoned this approach completely in RAxML.

However, the advent of gappy phylogenomic alignments, that is alignments that contain a large amount of structured (non-randomly distributed across the alignment) missing data regions per gene for the taxa under study, motivated us to re-assess SEVs in a simpler and thus more efficient setting.

**Figure 3:**
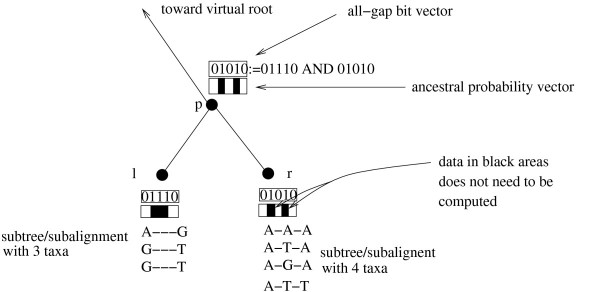
**Using Subtree Equality Vectors to save computations for all-gap alignment sites in subtrees**.

Gaps and undetermined characters are mathematically equivalent in the standard ML framework. Since structured patches of missing data dominate current phylogenomic datasets (typically the amount of missing data ranges between 50% to 90%), we only track subtree site patterns that entirely consist of gaps/undetermined characters (e.g., we are only interested in subtree sites of the from: ---- in a subtree of size 4). Thereby, we avoid the more complex task (see [20] and [21]) of tracking *all *identical subtree site patterns (e.g., detecting all sites of the form: ACCT in a subtree of size 4). This restriction simplifies the required bookkeeping procedure and data structure significantly, because we only need to know whether a subtree site consists entirely of gaps or not. Thus, given an alignment with *n *sites, it suffices to enhance the data structures for storing tips and inner nodes by a simple bit vector with *n *bits. If all-gap sites are represented by 1 and non-gap sites by 0, we simply need to execute a bit-wise AND on the respective bit vectors of the child nodes *l *and *r *in conjunction with the tree traversal for computing the likelihood to determine the all-gap sites at the ancestral node *p *(see Figure 3). We can then use this bit vector at *p *to determine if we need to compute something at a site *i *or not.

We have implemented this method for DNA and protein data under the Γ model of rate heterogeneity in RAxML v728 (alpha) available at http://wwwkramer.in.tum.de/exelixis/software.html. Evidently, the efficiency of this approach depends on the proportion of gaps/missing data and the distribution of gaps in the input alignment. Since areas of missing data are typically well-structured in current phylogenomic datasets, this approach is expected to work well with this kind of input data. To facilitate the deployment of the SEV-based version of the likelihood function, we have integrated an automatic performance test that decides whether to use the SEV-based or the standard likelihood implementation. When the starting tree has been computed or parsed by RAxML, the program will execute a full tree traversal (re-compute all ancestral probability vectors) for the standard *and *the SEV-based likelihood function implementation and measure the respective execution times. If the execution time of the SEV-based approach is 20% smaller than that of the standard implementation, RAxML will automatically use the SEV-based implementation for all subsequent likelihood computations. The threshold of 20% is based on empirical observations. While SEVs can speed-up ancestral probability vector computations, SEVs slightly slow down the branch length optimization and likelihood computation (at the root) functions because of the memory accesses to the bit vectors.

#### Saving Memory with SEVs

SEVs as implemented here, can also be deployed to reduce memory requirements. As mentioned above, if, at an ancestral node *p *we encounter an all-gap site, we completely omit its computation. In order to accomplish this, we need to maintain only one additional ancestral probability vector site, that contains the signal for all-gap sites. Consider an ancestral probability vector where 50% of the entries in the all-gap site bit-vector are set to 1, that is, where we only need to compute 50% of the ancestral probability vector entries with respect to the total alignment length.

**Figure 4:**
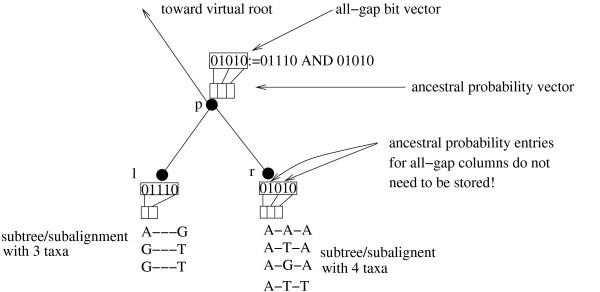
**Using Subtree Equality Vectors to save computations and memory for all-gap alignment sites in subtrees**.

We can observe that, in addition to saving 50% of the computations required for this ancestral probability vector, we can also save 50% of the memory space required for storing the ancestral probability vector (see Figure 4). Thus, the memory requirements for each ancestral node can be determined on-the-fly as we traverse the tree, by subtracting the number of entries that are set to 1 in the bit vector from the input alignment length. Remember that, the bit vectors we deploy are always as long as the input alignment.

The key technical problem that arises is that, the required ancestral probability vector lengths at inner nodes will change dynamically when the tree topology changes or even when the tree is just re-rooted. Given a rooting of the tree, one may think of this as ancestral probability vectors becoming longer while one approaches the root of the tree. At present we have implemented this by dynamically freeing and allocating memory (using free() and malloc()) at each ancestral node. The reallocation only takes place when the all-gap bit-vector count (number of bits set to 1) corresponding to the required ancestral probability vector does not equal the all-gap bit-vector count of the current ancestral probability vector at an ancestral node.

Note that, the concepts presented here can also be applied to phylogenomic datasets with joint branch length estimates across partitions, while the conceptually different ideas presented in [22] can only be applied to partitioned phylogenomic datasets with per-partition branch length estimates.

### Numerical Problems of the Γ Model of Rate Heterogeneity

Numerical scaling of the entries in the ancestral (inner) probability vectors during likelihood computations on trees, for avoiding numerical underflow has become a standard technique that is implemented in most likelihood-based programs (PHYML, Mr-Bayes, GARLI, RAxML, BEAST, etc.). For an overview of numerical scaling techniques, please refer to [16]. A numerical problem that arises for very large trees with more than approximately 50,000 taxa in RAxML (and probably all other likelihood-based programs as well) is associated with the widely used [23] Γ model of rate heterogeneity [14].

For the Γ model, a discrete approximation (typically using 4 discrete rates) is used to approximate the integral of the likelihood over the Γ curve at each site. That is, instead of computing the ancestral probabilities *L*(*A*), *L*(*C*), *L*(*G*), *L*(*T*) for the 4 nucleotides A, C, G, T at a specific site of an ancestral node in the tree (see Equation 1), one needs to compute those probabilities for all 4 discrete Γ rates *r*_0_, *r*_1_, *r*_2_, *r*_3_. Thus, every site of an ancestral probability vector comprises 16 values:

(2) $$\begin{array}{c} L{\left(A\right)}_{r_{0}},L{\left(C\right)}_{r_{0}},L{\left(G\right)}_{r_{0}},L{\left(T\right)}_{r_{0}},...\\ \;\;\;\;\;...,L{\left(A\right)}_{r_{3}},L{\left(C\right)}_{r_{3}},L{\left(G\right)}_{r_{3}},L{\left(T\right)}_{r_{3}}. \end{array}$$

The numerical problem that arises with the Γ model on very large trees is that those 16 values need to be jointly scaled numerically (all 16 values are multiplied by a large number) to avoid numerical underflow (see below).

Scaling of the probability vector entries may be conducted as follows: At a specific site *c *of an ancestral probability vector for DNA data $\vec{L}$ we scale the entries if, for instance,

(3) $$\begin{array}{c} L{\left(A\right)}_{r_{0}}\left(c\right)<\varepsilon \wedge L{\left(C\right)}_{r_{0}}\left(c\right)<\varepsilon ,...\\ \;\;\;\;\;\;...,L{\left(G\right)}_{r_{3}}\left(c\right)<\varepsilon \wedge L{\left(T\right)}_{r_{3}}\left(c\right)<\varepsilon \end{array}$$

where *ε *can be set to *ε *:= 1/2^256 ^under double precision arithmetics. Thus, we decide to scale up all ancestral probability vector entries at a site *c*, when *all *unscaled entries for *all *discrete rates are smaller than some pre-defined *ε*.

Other options for scaling exist. For instance, one calculates a scaling factor such that the largest of the 16 ancestral probability values at a site is scaled to 1.0. One can also conduct this type of scaling at every ancestral probability vector without checking that all values are smaller than some *ε*. We also experimented with such alternative implementations for numerical scaling in RAxML, but were not able to solve the general scaling problem for the Γ model of rate heterogeneity (see below). With alternative scaling implementations, the fundamental numerical problem occurred again for slightly larger tree sizes.

If according to Equation 3 a probability vector column *c *at vector $\vec{L}$ needs to be scaled, we simply multiply all entries

(4) $${\vec{L_{A}}}_{r_{0}}\left(c\right),{\vec{L_{C}}}_{r_{0}}\left(c\right),...,{\vec{L_{G}}}_{r_{3}}\left(c\right),{\vec{L_{T}}}_{r_{3}}\left(c\right)$$

by 2^256^.

In order to correct (undo) the scaling multiplications (accumulated during a tree traversal) at the virtual root, we need to keep track of the total number of scaling operations conducted per column. For this, we use integer vectors $\vec{U}$ that maintain the scaling events and correspond to the respective probability vectors at inner nodes. As we traverse the tree to compute an ancestral vector ${\vec{L}}^{\left(k\right)}$ from two child vectors ${\vec{L}}^{\left(i\right)}$ and ${\vec{L}}^{\left(j\right)}$ the scaling vector is initially updated as follows ${\vec{U}}^{\left(k\right)}\left(c\right):={\vec{U}}^{\left(i\right)}\left(c\right)+{\vec{U}}^{\left(j\right)}\left(c\right)$. Then, if an entry of ${\vec{L}}^{\left(k\right)}$ needs to be scaled at position *c *we increment ${\vec{U}}^{\left(k\right)}\left(c\right):={\vec{U}}^{\left(k\right)}\left(c\right)+1$. The scaling vectors at the tips of the tree are not allocated, but implicitly initialized with 0.

At the virtual root, given ${\vec{L}}^{\left(i\right)}$, ${\vec{L}}^{\left(j\right)}$ and the corresponding scaling vectors ${\vec{U}}^{\left(i\right)}$, ${\vec{U}}^{\left(j\right)}$, we can compute the likelihood under the Γ model as follows:

(5) $$l\left(c\right)=\varepsilon^{U^{\left(i\right)}\left(c\right)+U^{\left(j\right)}\left(c\right)}\left(\frac{1}{4}\cdot Q_{r_{0}}+\frac{1}{4}\cdot Q_{r_{1}}+\frac{1}{4}\cdot Q_{r_{2}}+\frac{1}{4}\cdot Q_{r_{3}}\right)$$

where, for instance,

(6) $$Q_{r_{0}}:=\left(\overset{T_{r_{0}}}{\underset{R=A_{r_{0}}}{\sum }}\left(\pi_{R}{\vec{L}}_{R_{r_{0}}}^{\left(i\right)}\left(c\right)\overset{T_{r_{0}}}{\underset{S=A_{r_{0}}}{\sum }}P_{RS}\left(b_{vr}\right){\vec{L}}_{S_{r_{0}}}^{\left(j\right)}\left(c\right)\right)\right)$$

If we take the logarithm of *l*(*c*) this can be rewritten as:

(7) $$\begin{array}{c} log\left(l\left(c\right)\right)=\left(U^{\left(i\right)}\left(c\right)+U^{\left(j\right)}\left(c\right)\right)log\left(\varepsilon \right)+...\\ \;\;\;+log\left(\frac{1}{4}\cdot Q_{r_{0}}+\frac{1}{4}\cdot Q_{r_{1}}+\frac{1}{4}\cdot Q_{r_{2}}+\frac{1}{4}\cdot Q_{r_{3}}\right) \end{array}$$

As can be observed, if all 16 values are scaled jointly, the scaling multiplications can be easily undone numerically at the virtual root, when the overall likelihood of the tree is computed (see [16] for more details). This is not the case, if one intends to scale the ancestral probability values individually on a per-rate (*r*_0_, ..., *r*_3_) basis.

The problem that arises with using Γ on very large trees is that, the 16 ancestral probability values (using four discrete Γ rates for DNA data), may have such highly divergent numerical values because of the 4 discrete rates *r*_0_, *r*_1_, *r*_2_, *r*_3_, that scaling across all 16 of them will still not prevent numerical under-flow. In other words, the smallest value of those 16 will be too small and the largest too large to fit into the representable machine number range between 0.0 and 1.0. Scaling values above 1.0 will yield numerical overflow and does hence not provide a solution either. At present, we are not aware how scaling multiplications can be undone (reversed) in a numerically stable way at the root, if one scales the probability values for each discrete rate category individually. This phenomenon could also occur for models other than Γ that only use four values per site (see below), but will probably occur on significantly larger trees. While one could use extended precision libraries, as provided for instance by the GNU Scientific Library, the negative performance impact will be such, that the computation of large trees also becomes prohibitive.

It is however possible to address this scaling problem by discarding the per-rate/category likelihoods that contribute least to the overall likelihood and thereby approximate the GAMMA-based likelihood score of a site. This approach (currently unpublished) has been implemented in PhyML [5].

To this end, we advocate the usage of per-site rate categories as proposed and implemented in RAxML (CAT approximation of rate heterogeneity [24]), PhyloBayes [25], or FastTree 2.0 [6]. While methods that model among-site rate heterogeneity by using one rate per site can help to significantly reduce computational requirements (memory utilization and floating point operations are reduced by approximately a factor of four compared to a Γ model with four discrete rates [24]), they can also help to resolve the aforementioned numerical problems with Γ on very large trees. Note that, the CAT approximation as implemented in RAxML should not be confused with the substantially different CAT model implemented in PhyloBayes. The unfortunate fact that an identical acronym is used is because at time of publication, the author of RAxML was not aware of the PhyloBayes CAT model that was introduced earlier.

One key issue with the so-called CAT approximation of rate heterogeneity [24] in RAxML was that branch length values were meaningless. This has been corrected in RAxML version 7.2.9 (available at http://wwwkramer.in.tum.de/exelixis/software/RAxML-7.2.9.tar.bz2) by appropriately re-scaling the per-site rate categories such that the mean substitution rate is 1.0. While, as we show, the correctly scaled CAT-based branch lengths are highly correlated (see results section) with the branch lengths obtained from the Γ model, the overall tree length obtained for Γ and CAT-based branch lengths can vary significantly. Analogous results were obtained for FastTree 2.0 [6]. This does not represent a problem as long as post-analysis tools for trees (e.g, divergence-time estimation, ancestral state reconstruction) do not rely on absolute branch length values.

Another issue that needs to be addressed is that CAT-based log likelihood scores across different runs (e.g., two ML searches on the original alignment using different starting trees) can not be compared directly, because the estimates and assignments of rate categories to sites may be slightly different for each search/tree topology. Therefore, for comparing likelihood scores of trees under the CAT approximation of rate heterogeneity, we need to score all alternative trees under the same assignment of rate categories to sites. RAxML v729 implements the -f n option to score a set of fixed trees under the same rate category to site assignment.

Finally, one also needs to assess how differently trees are ranked (ordered) with respect to their log likelihood scores, if scored under Γ or under CAT. While one would not expect a perfect rank correlation (because CAT has more ML model parameters than Γ), because both models accomodate rate heterogeneity, the correlation should not be too low either.

## Results and Discussion

### Test Datasets

To assess our methods, we used two large multi-gene datasets of plants.

The first dataset comprises 37,831 taxa and 9,028 sites and was obtained as follows: We assembled a DNA sequence matrix of 37,831 seed plant taxa consisting of the chloroplast regions atpB (1,861 taxa, > 2.6 Megabases [Mb]), matK (10,886 taxa, > 14.3 Mb), rbcL (7,319 taxa, > 9.7 Mb), trnK (4,163 taxa, > 7.5 Mb), and trnL-trnF (17,618 taxa, > 13 Mb), and the internal transcribed spacer (ITS; 26,038 taxa, > 14.3 Mb), using the Phylogeny Assembly with Databases tool (PHLAWD [12]http://code.google.com/p/phlawd). All sequence alignments were conducted using MAFFT version 6 [26] for initial alignments and MUSCLE for profile alignments [27]. Alignment matrix manipulations were performed with Phyutility [28].

The second dataset comprises 55,593 taxa and 9,853 sites and was obtained using the same pipeline as described above. The gene regions used were atpB (2,346 taxa, > 3.6 Megabases [Mb]), matK (14,848 taxa, > 33.6 Mb), rbcL (10,269 taxa, > 14.9 Mb), trnK (5,859 taxa, > 15.3 Mb), and trnL-trnF (25,346 taxa, > 30.1 Mb), and the internal transcribed spacer (ITS; 37,492 taxa, > 30.9 Mb).

For ease of reference we henceforth denote the 37,831 taxon datasets as 38 K and the 55,593 taxon as 56 K. Trees computed on the 56 K dataset have recently been published [29] and the alignment is available at http://datadryad.org/. The 38 K dataset is currently unpublished, but will be made available immediately upon publication.

### Backbone Algorithm

To test the backbone algorithm we executed the dedicated RAxML version (available at http://wwwkramer.in.tum.de/exelixis/software/BackboneSearch.zip) with the experimental -L command line option. This option initially builds a backbone tree and then deploys the CAT approximation of rate heterogeneity [24] with the standard RAxML hill-climbing search algorithm [18,30] to apply lazy SPR moves (see [18]) within the backbone only. We used tree size reduction factors of 0.25 and 0.5. As starting trees, we used randomized stepwise addition order parsimony starting trees generated with RAxML v727 (-y option). For each dataset, we inferred 10 ML trees for each of the 10 parsimony starting trees. RAxML was executed using the Pthreads-based parallel version [31] with 16 threads on unloaded Quad-Core AMD Opteron nodes with 16 cores and 128 GB RAM each.

We computed average runtimes over 10 runs for the 38 K and 56 K datasets respectively. For each backbone tree, we also computed the theoretical minimum number of bytes (denoted as *Memory for Backbone*) required to store the ancestral probability vectors at the virtual tips and the inner nodes which dominate memory requirements. If the branch length optimization process, unlike in our current implementation, is limited to optimizing branches within the backbone, this theoretical minimum value represents a good estimate of the memory footprint for a backbone tree search. We also computed the respective memory requirements for the comprehensive tree (denoted as *Memory for Full tree*), which reflects the 'standard' memory requirements when no reduction factor is applied.

These values (see Tables 2 and 3) provide a notion of the potential memory savings that can be achieved by the backbone approach. In Tables 2 and 3 we also provide the respective execution times and average log likelihood scores obtained by using the backbone algorithm (*R *:= 0.25, *R *:= 0.5) and a comprehensive search on the full tree (*R *:= 1.0). Those values have been averaged over 10 runs (10 starting trees). While execution times can be reduced by the backbone approach, log likelihood scores obtained by conducting searches on a backbone are slightly worse than those obtained by searching on the full tree. Also note that, for unfavorable tree shapes, that is, tree shapes where a substantial part of the phylogenetic signal is located at or near the tips, a too aggressive setting of *R *may potentially generate unfavorable results since this signal can be lost in the backbone. However, some exploratory tests with simulated data (results not shown) did not show such an effect for backbone searches.

**Table 2:** Average runtimes, memory requirements, and log likelihood scores (over 10 runs) for the 38 K dataset

37831 taxa			
	**R = 0.25**	**R = 0.5**	**R = 1**

Runtime (h)	30.41	38.60	54.03

Memory for Backbone (GB)	4.90	7.70	N/A

Memory for Full tree (GB)	10.33	10.33	10.33

LogLikelihood (Avg)	-5531436	-5530051	-5529406

LogLikelihood (Std Dev)	943.26	770.47	1307.16

Avg (logLH - logLH(R = 1))	2030.24	645.31	0.0

**Table 3:** Average runtimes, memory requirements, and log likelihood scores (over 10 runs) for the 56 K dataset

55593 taxa			
	**R = 0.25**	**R = 0.5**	**R = 1**

Runtime (h)	50.17	63.22	85.89

Memory for Backbone (GB)	8.22	12.72	N/A

Memory for Full tree (GB)	16.82	16.82	16.82

LogLikelihood	-7063342	-7061516	-7060488

LogLikelihood (Std Dev)	1727.90	1761.27	1718.47

Avg (logLH - logLH(R = 1))	2853.41	1028.04	0.0

**Figure 5:**
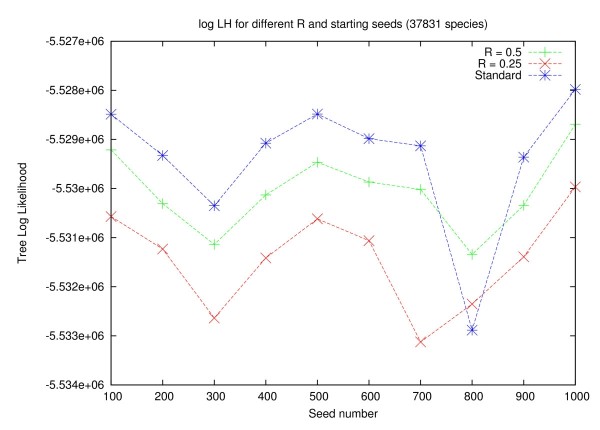
**Log Likelihood scores for different Reduction factors (38 k dataset)**. Plot of log likelihood scores under GTR+Γ of the final trees obtained by each method as a function of the starting tree (random number seed) for the 38 K dataset. Each LH score (point) results from an independent search. The lines linking the points are only guiding the eye.

**Figure 6:**
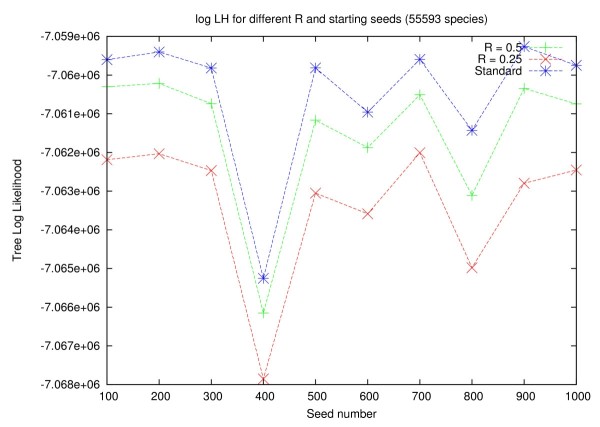
**Log Likelihood scores for different Reduction factors (56 k dataset)**. Plot of log likelihood scores under GTR+Γ of the final trees obtained by each method as a function of the starting tree (random number seed) for the 56 K dataset. Each LH score (point) results from an independent search. The lines linking the points are only guiding the eye.

In Figures 5 and 6 we show that the choice of the random number seed (-p option in RAxML), that determines the shape of the starting trees, has a significant impact on the final log likelihood score (computed under GTR+Γ), irrespective of the search strategy that is used. On average, searches on the full tree yield better likelihood scores than searches on backbone trees. However, the variance of the likelihood score as a function of the starting tree (parsimony random number seed) is analogous to the score variance between full and backbone tree searches. For example, on the 38 K dataset, the log likelihood scores on 10 final trees obtained for full searches show a standard deviation of 1307 log likelihood units. The average difference in log likelihood scores per starting tree between the full search and a backbone search with *R *:= 0.50 is only 645 log likelihood units and 2030 log likelihood units for backbone searches with *R *:= 0.25, respectively.

Given the runtimes savings that can be achieved by the backbone approach, backbone tree searches can be used, for instance, to explore a larger number of parsimony starting trees which substantially influence the final log likelihood scores. A reasonable strategy for finding best-known ML trees may consist in starting many fast searches with a relatively aggressive setting of *R *:= 0.25 to identify/determine a set of 'good' starting trees that yield the best final log likelihood scores. In a second step, full tree searches can be conducted on those promising starting trees to find trees with even better scores.

We used simulated datasets in order to better understand the impact of the backbone algorithm on topological accuracy. We ran indelible [32] to generate simulated MSAs of 1500 taxa (575 bp) and 5000 taxa (1074 bp). We compared the symmetric difference (number of bipartitions that differ between two topologies) between the true tree and the topologies from the starndard full search and the backbone-based ones. For each dataset, the full search and the backbone search with *R *:= 0.25 and *R *:= 0.5 were ran 5 different times with different starting trees. Table 4 shows the average symetric differences among all approaches for the dataset with 1500 taxa. We see that, in terms of topological accuracy, applying the reductions of *R *:= 0.25 and *R *:= 0.5 yield topologies that remain close to the standard full search. Furthermore, the distance to the true tree is not increased by the reduction.

**Table 4:** Average symmetric differences (over 5 runs) for the 1500 dataset

Average Symmetric Difference			
	***R *:= 0.25**	***R *:= 0.5**	***R *:= 1**

*R *:= 0.25	182.6	169.9	188.0

*R *:= 0.5	169.9	152.8	146.2

*R *:= 1	188.0	146.2	133.0

True Tree	398.8	382.0	388.0

The likelihood scores for both simulated datasets follow the same pattern as in the case of real data. These results, details on how the simulation datasets were generated, as well as the symetric difference for the 5000 taxa dataset have been included in the additional file 1.

### SEV Performance

We also used the 38 K and 56 K datasets to test memory savings and speedups achieved by applying the adapted SEV technique to phylogenomic datasets. The gappyness (percentage of missing data in the alignments) is 81.53% for 38 K and 83.40% for 56 K, respectively.

For each alignment, we computed a parsimony starting tree with RAxML that was then evaluated (model parameter and branch length optimization without tree search, RAxML -f e option) with RAxML under the GTR+Γ model using the SEV reimplementation (with and without memory saving) and using the standard likelihood implementation.

The standard implementation required 41 GB of memory on the 38 K dataset and 66 GB of memory on the 56 K dataset. The SEV technique with the memory saving option enabled (-U option, available as of RAxML v727) reduced memory footprints under Γ to 14 GB (38 K) and 21 GB (56 K) respectively. The log likelihood scores for all three implementations were exactly identical. As shown in Tables 5 and 6, the runtimes of the SEV-based versions are 25-40% faster than for the standard implementation. The runtime differences between the SEV-based implementation with memory saving enabled and the plain SEV version without memory saving, can be attributed to differences in memory access patterns. While both versions conduct the same number of computations, the memory-saving version needs to make millions of calls to OS routines (free() and malloc()) while the plain SEV version exhibits a higher memory footprint and thereby, potentially, a higher cache miss rate.

**Table 5:** SEV evaluation for the 38 k dataset

37831 taxa			
	**SEVs**	**SEVs with memory saving**	**standard**

Runtime (s)	4125.1	4116.8	6541.1

Memory (GB)	42	15	41

LogLikelihood	-5528590	-5528590	-5528590

Execution times and memory requirements for optimizing model parameters and branch lengths under on the 38 K dataset using SEVs, SEVs with memory saving, and the standard likelihood implementation.

**Table 6:** SEV evaluation for the 56 k dataset

55593 taxa			
	**SEVs**	**SEVs with memory saving**	**standard**

Runtime (s)	7145.2	8095.1	11181.4

Memory (GB)	67	29	67

log likelihood	-7059556	-7059556	-7059556

Execution times and memory requirements for optimizing model parameters and branch lengths under on the 56 K dataset using SEVs, SEVs with memory saving, and the standard likelihood implementation.

### Estimating branch lengths and computing likelihood scores with CAT

We optimized branch lengths and model parameters under CAT and Γ using RAxML v730 (-f n option) on collections of 32 and 22 final ML trees for the 38 K and 56 K partitioned datasets, respectively. For the CAT model, we also assessed the impact of using, 8, 16, 25 (default), and 40 per-site rate categories.

For each tree, we computed the average Pearson correlation coefficient between the branch lengths obtained under CAT (for 8, 16, 25, and 40 per-site rate categories) and the branch lengths as estimated under Γ. We also computed the average tree length ratio over all 32 trees.

To determine if the trees are ranked in the same order by their respective Γ and CAT log likelihood scores, we computed the Spearman rank correlation of the CAT- and Γ-based tree rankings. As shown in Tables 7 and 8, the Spearman correlation was above 0.99 in all cases. This indicates that, trees are ordered in almost the same way, regardless of whether they are scored under CAT or Γ.

**Table 7:** Correlations between CAT and Γ models for the 38 k dataset

37831 taxa, 32 ML trees				
Number of per-site rate categories	8	16	25	40

Average BL correlation with Γ	0.994	0.995	0.995	0.995

Average Tree length ratio (Γ/CAT)	1.743	1.739	1.739	1.739

Spearman rank correlation(Γ, CAT)	0.994	0.992	0.992	0.992

Correlation between CAT and Γ-based ML branch length estimates, total tree length ratios, and Spearman rank correlation coefficients between likelihood-induced tree rankings obtained from CAT and Γ for dataset 38 K.

**Table 8:** Correlations between CAT and Γ models for the 56 k dataset

55593 taxa, 22 ML trees				
Number of per-site rate categories	8	16	25	40

Average BL correlation with Γ	0.877	0.877	0.877	0.877

Average Tree length ratio (Γ/CAT)	1.569	1.567	1.567	1.608

Spearman rank correlation(Γ, CAT)	1.0	1.0	1.0	1.0

Correlation between CAT and Γ-based ML branch length estimates, total tree length ratios, and Spearman rank correlation coefficients between likelihood-induced tree rankings obtained from CAT and Γ for dataset 55 K.

We also used FastTree 2 (with options -gamma -nt -nome -mllen) to score both collections of trees under the hybrid CAT/Gamma20 model [6]. Once again, the Spearman rank correlation of FastTree CAT/Gamma20 and RAxML Γbased tree rankings remained above 0.99 in all cases.

The average branch length correlation between CAT and Γ optimized branches was above 0.87. On those two large datasets, the absolute length of Γ-based branch length estimates was larger than for CAT as shown by the average tree length ratios.

We also executed analogous analyses on 10 smaller single-gene (and non-partitioned) datasets with 1481 up to 4114 taxa. In addition, we evaluated significantly larger ML tree collections (160 ML trees per dataset) for those smaller datasets. These additional experiments confirmed our observations for the 38 K and 55 K datasets and also revealed that the total tree length under CAT, can also be larger than the Γ-based tree length. Respective plots for all datasets are provided in the additional file 1.

## Conclusions

We have explored several techniques, addressed problems, and proposed some solutions for phylogenetic tree inference with likelihood-based methods on trees with several tens of thousands of taxa.

Initially, we revisit and re-assess techniques for reducing the tree size, inspired by earlier work on a program called Phylogenetic Navigator (Phy-Nav). Significant effort was invested in exploring different backbone construction techniques (results/experiments not shown). Here, we describe the method that worked best with respect to final log likelihood scores. Such backbone-based techniques can help to reduce memory footprints and execution times. However, in almost all cases they yield final trees with worse likelihoods compared to comprehensive tree searches on a full, unreduced tree. We find that likelihood scores of final trees heavily depend on the respective starting trees, and conclude that backbone approaches can be deployed for identifying 'good' starting trees, that can then be further refined using a comprehensive tree search.

We have adapted and re-implemented the SEV technique for phylogenomic datasets with missing data and enhanced it by a novel memory-saving option. This new technique, is generally applicable to all likelihood-based codes and can reduce execution times by 25-40% on sufficiently 'gappy' datasets by omitting redundant computations. More importantly, the revised SEV technique can be deployed to achieve significant memory savings that are almost proportional to the amount of missing data in the test datasets. This technique has already been fully integrated into the standard RAxML distribution (as of v727). Moreover, RAxML will automatically determine whether to use the standard likelihood implementation or the SEV-based likelihood implementation.

Finally, we analyze problems associated to numerical scaling for avoiding underflow, that can occur when using the Γ model of rate heterogeneity on very large datasets. While for the 38 K and 55 K datasets we were still able to evaluate trees under Γ, on some even larger datasets that we are currently analyzing (e.g., 116,408 taxa 18,692 sites) numerical scaling under Γ appears to be impossible using 64-bit floating point arithmetics. To this end, we advocate the usage of models that rely on per-site rate categories for accommodating rate heterogeneity among sites. Clearly, further research is required in this area to devise statistically robust and meaningful models. Nonetheless, we provide an empirical assessment of branch length estimates as obtained under Γ and the RAxML-specific implementation for estimating and assigning per-site evolutionary rate categories. We find that, given proper scaling of per-site rates, branch lengths between CAT and Γ based trees are highly correlated, despite the fact that absolute branch length values can differ substantially. We also find that, ordering tree collections using Γbased and CAT-based log likelihood scores induces very similar rankings of trees as determined be the Spearman rank correlation coefficient.

The work presented here has a clear exploratory flavor and we hope that it will be useful to the community for identifying future research directions pertaining to large-scale phylogenetic inference using likelihood-based methods. The problems and solutions we discuss in this paper, emerged within the framework of the plant tree of life grand challenge project that aims at reconstructing the plant tree of life comprising approximately 500,000 taxa.

## Authors' contributions

FIC developed and implemented the backbone algorithm and conducted all computational experiments. AS developed and implemented the SEV-based techniques and implemented the CAT re-scaling procedure. SAS assembled the large test datasets. FIC, AS, and SAS wrote and edited the manuscript.

## Supplementary Material

Additional file 1 **Supplementary Material**. Assesment of alternative criteria to identify the innermost node of a tree. Evaluation of the backbone algorithm with simulated data: Simulation details and symmetric difference for the 5000 taxa dataset, log likelihood scores for ML trees on simulated datasets(1500 and 5000 species). Evaluation of the backbone algorithm with real data and comparison with FastTree 2. Correlation between CAT and Γ-based ML branch length estimates, total tree length ratios, and Spearman rank correlation coefficients between likelihood-induced tree rankings obtained from CAT and Γ for 12 different datasets ranging from 1481 up to 4114 number of taxa. Correlations between log likelihood scores under the RAxML CAT model and Γ model for the 38 k and 56 k dataset. Correlations between log likelihood scores under the RAxML CAT model and the FastTree 2 CAT/Gamma20 model for the 38 k and 56 k dataset. Click here for file
