# Supplementary material for: Algorithms, data structures, and numerics for likelihood-based phylogenetic inference of huge trees
Source: BMC Bioinformatics. 2011 Dec 13;12:470. doi: 10.1186/1471-2105-12-470 (PMC3267785; doi:10.1186/1471-2105-12-470)

Suplementary Material

Assesment of alternative criteria (to the default used) to identify the innermost node of tree.

In the next two tables, we provide the raw and normalized node distances between respective innermost nodes for trees with 37,831 taxa (average tree diameter: 164.3 nodes) and 55,593 taxa (average tree diameter: 186.0 nodes). For both datasets, all distances have been averaged across 10 different starting trees.

The tree diameter is defined as the number of nodes on the longest path between any pair of tips. We define the raw node distance between two nodes as the number of nodes on the path that connects the nodes.
The normalized node distance is defined as the raw node distance between alternative innermost nodes, divided by the tree diameter.

Alternative criterion	node -distance	Normalized node-distance
Lowest subtree length variance	0.000	0.000
Lowest node-to-tip distance variance	4.100	0.025
Maximal minimum node-to-tip distance	18.700	0.115

Table 1: Node-distances from the default criterion (37,831 taxa, averaged across 10 trees)} Several criteria can be employed to select the innermost node of an unrooted tree. The alternative innermost nodes are located close to each other with respect to the tree diameter (164.3)

Alternative criterion	node -distance	Normalized node-distance
Lowest subtree length variance	0.000	0.000
Lowest node-to-tip distance variance	2.500	0.013
Maximal minimum node-to-tip distance	11.900	0.064

Table 2: Node-distances from the default criterion (55,593 taxa, averaged across 10 trees)} Several criteria can be employed to select the innermost node of an unrooted tree. The alternative innermost nodes are located close to each other with respect to the tree diameter (186.0)

Evaluation of backbone algorithm with simulated data

All simulated datasets were generated with indelible using the following configuration:

```
[TYPE] NUCLEOTIDE 1 //  nucleotide simulation using algorithm from method 1

[MODEL] simple_model
  [submodel]  GTR 0.2 0.4 0.6 0.8 1.2 //  GTR: a=0.2, b=0.4, c=0.6, d=0.8, e=1.2, f=1
  [statefreq] 0.20 0.25 0.25 0.30         //  pi_T, pi_C, pi_A, pi_G
  [indelmodel]  NB 0.5 1
  [insertrate]  0.001
  [deleterate]  0.001

[TREE] random_tree
  [unrooted] 1500 2.4 1.1 0.2566 0.34  // ntaxa birth death sample mut
  [seed] 2381242

[PARTITIONS] simulated_gene
  [random_tree simple_model 400] // #base pairs

[EVOLVE] simulated_gene 1 simulated_alignment
```

For further details on the configuration file of indelible can be found in:
http://abacus.gene.ucl.ac.uk/software/indelible/tutorial/

	**R=0.25**	**R=0.5**	**R=1**
**R=0.25**	738.2	721.3	752.1
**R=0.5**	721.3	650.2	582.8
**R=1**	752.1	582.8	595.0
**True Tree**	1456.0	1395.2	1376.4

Table 3: Average symmetric differences (over 5 runs) for the 5000 taxa simulated dataset

Likelihood scores for ML trees based on different starting trees:

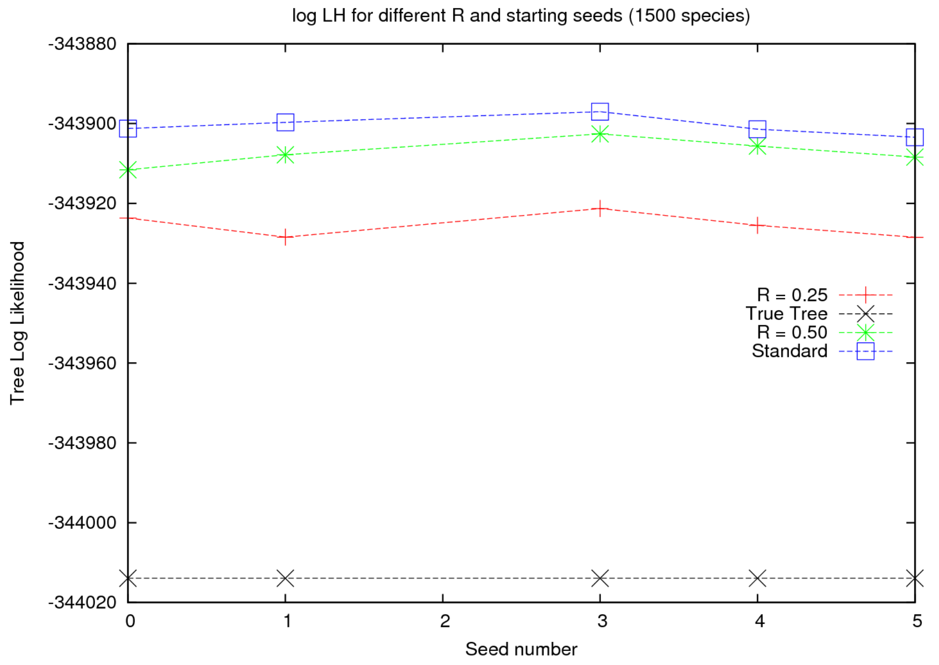

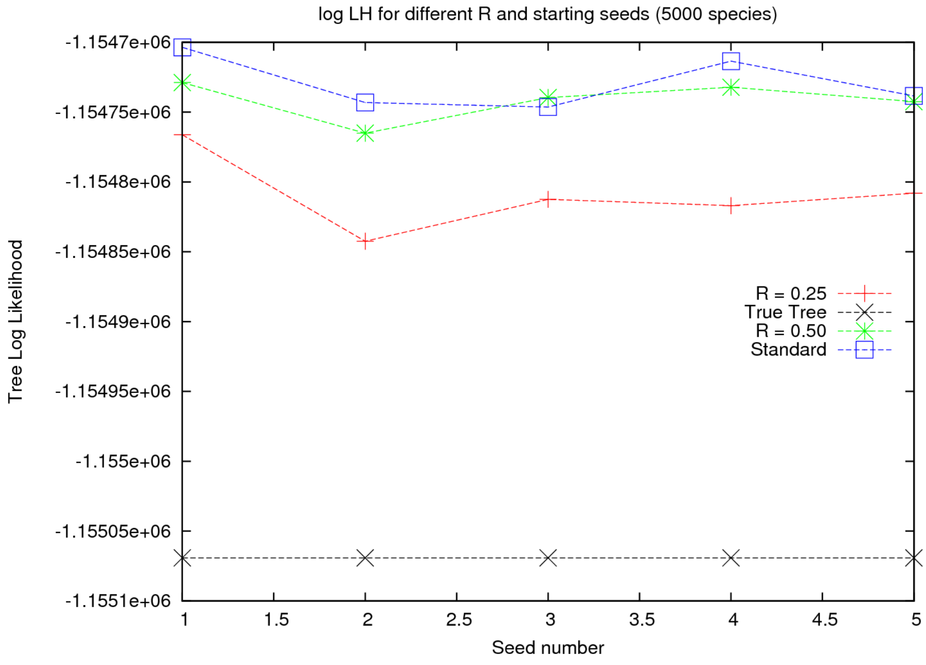

Evaluation of backbone algorithm with real data and comparison with FastTree 2

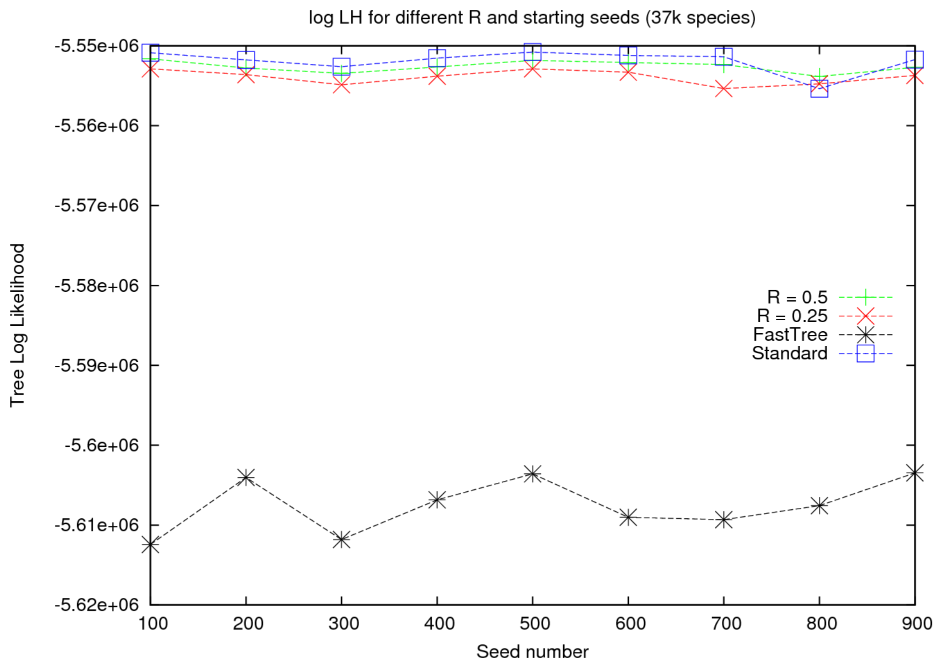

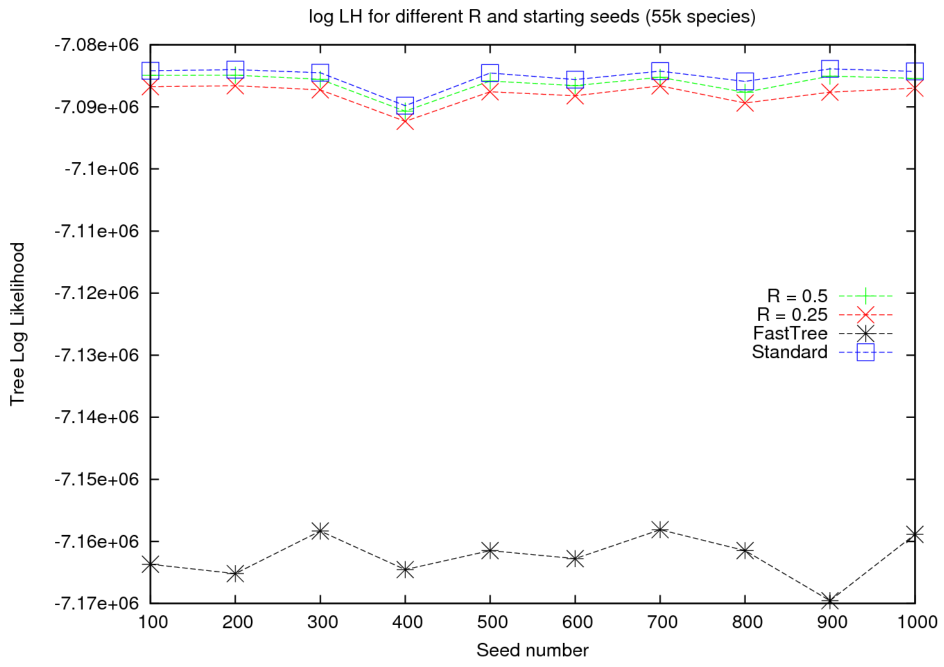

CAT and GAMMA correlations.

Each plot represents a bunch of 32 trees for a particaular dataset, e.g., LH1481 is a dataset comprising 1481 taxa.

1. The x axis represents log likelihood values of the evaluated tree under the GTR-GAMMA model
2. The y axis represents log likelihood values of the evaluated tree under the GTR-CAT model. The number of categories used is indicated in the label
3. rho is the Spearman rank correlation
4. tl is the average tree length ratio TL(GAMMA) / TL(CAT)
5. bl is the average branch-length correlation of the 32 trees (for each tree we computed the correlation of the branch lengths between the CAT and the GAMMA trees)

Correlations using the CAT model with 8 categories

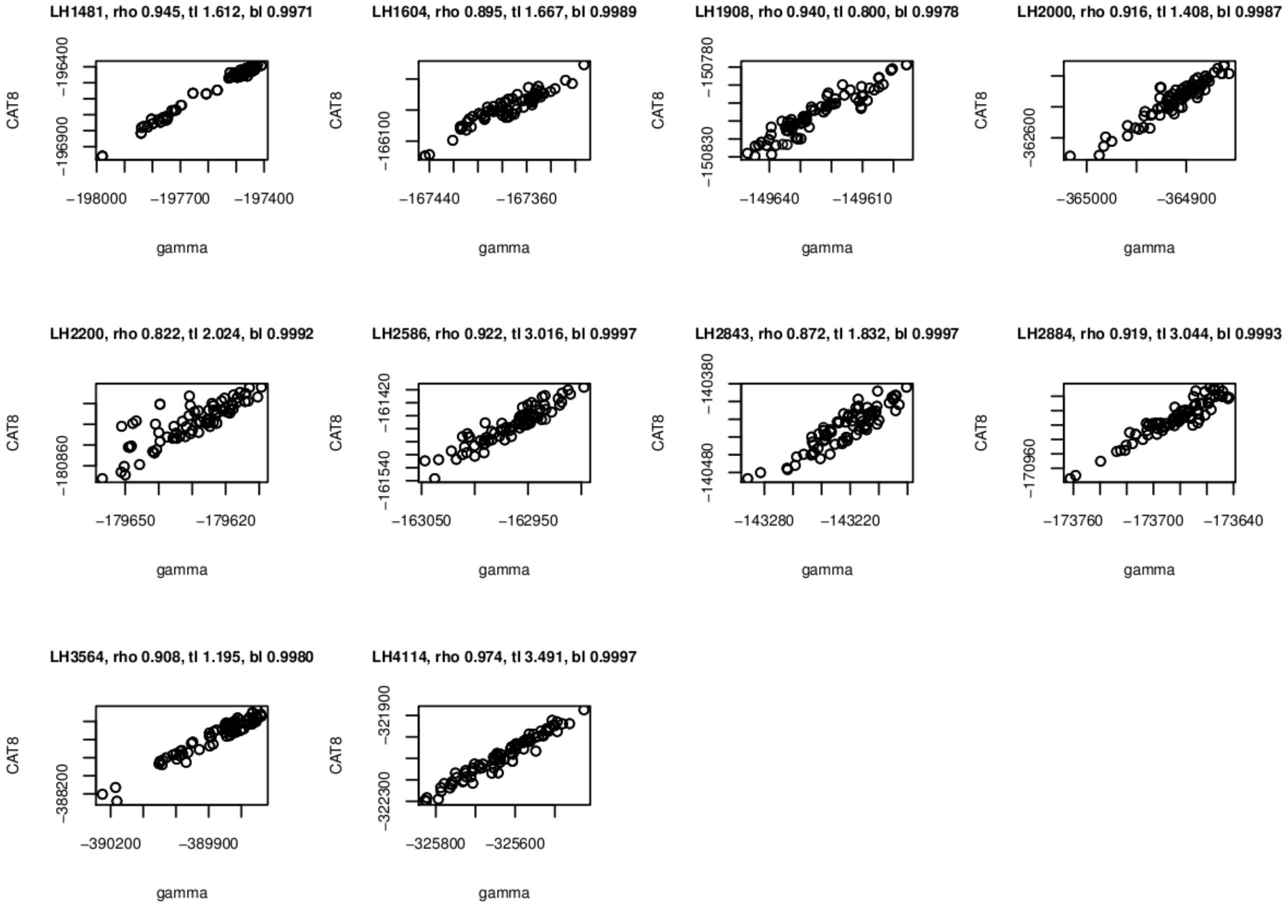

Correlations using the CAT model with 16 categories

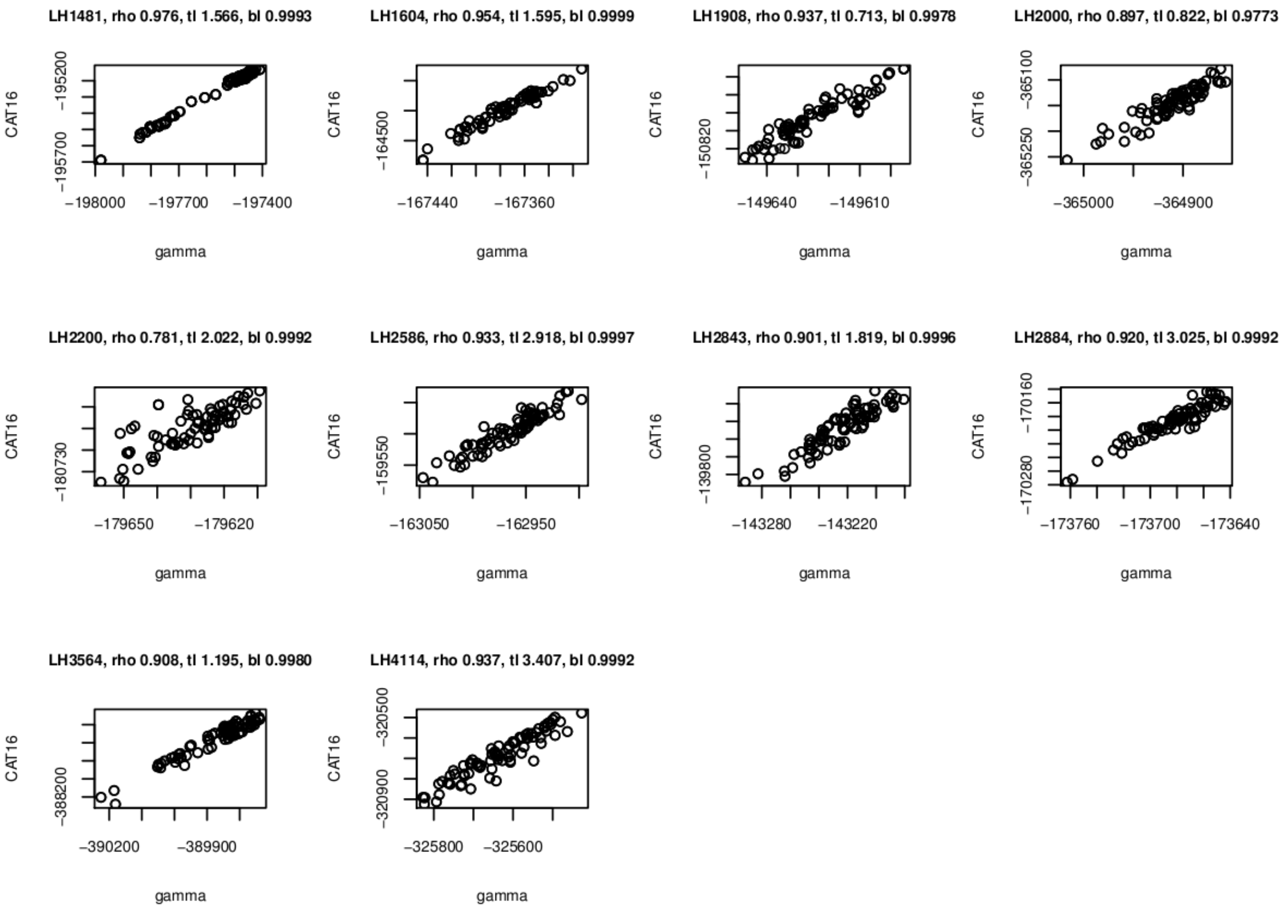

Correlations using the CAT model with 25 categories

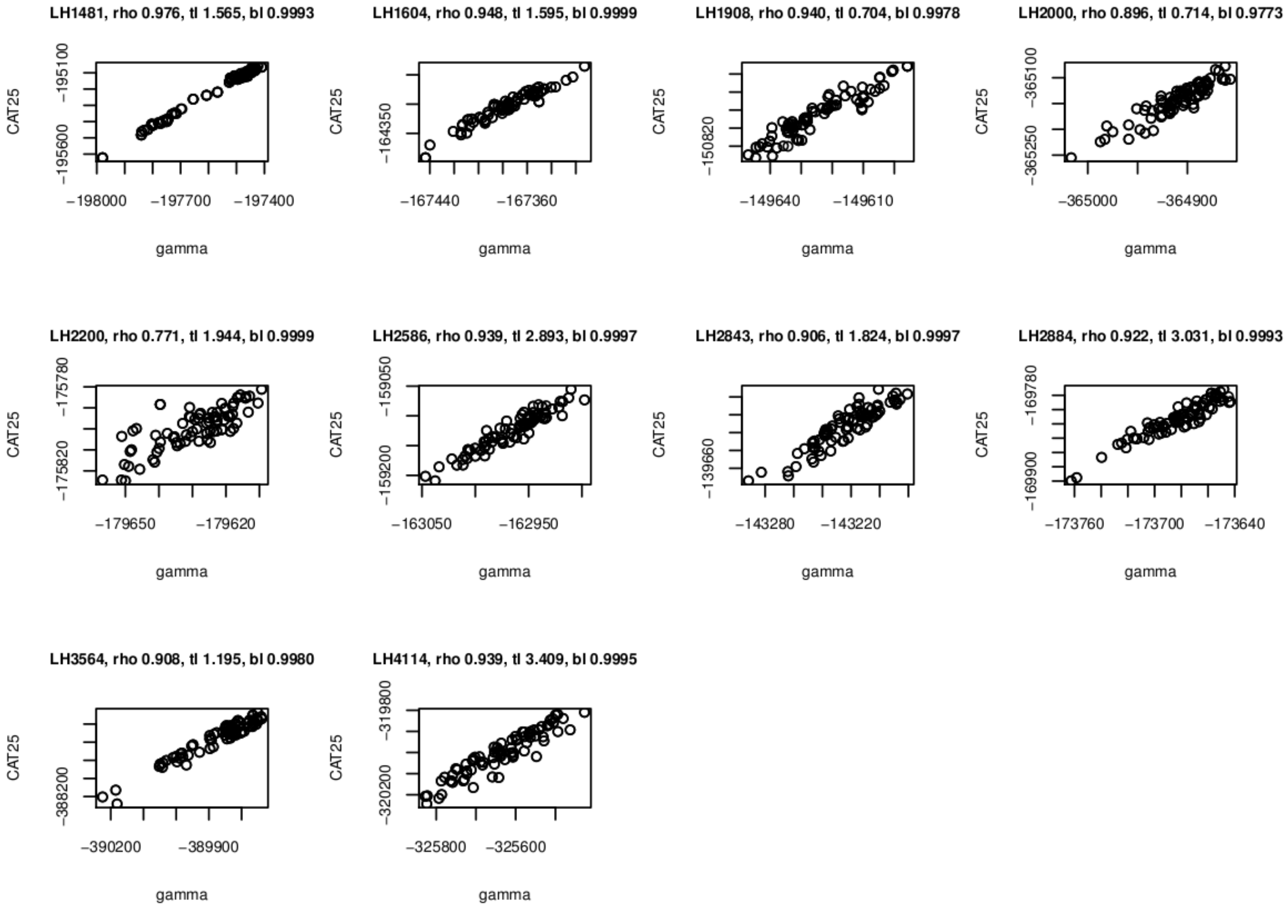

Correlations using the CAT model with 40 categories

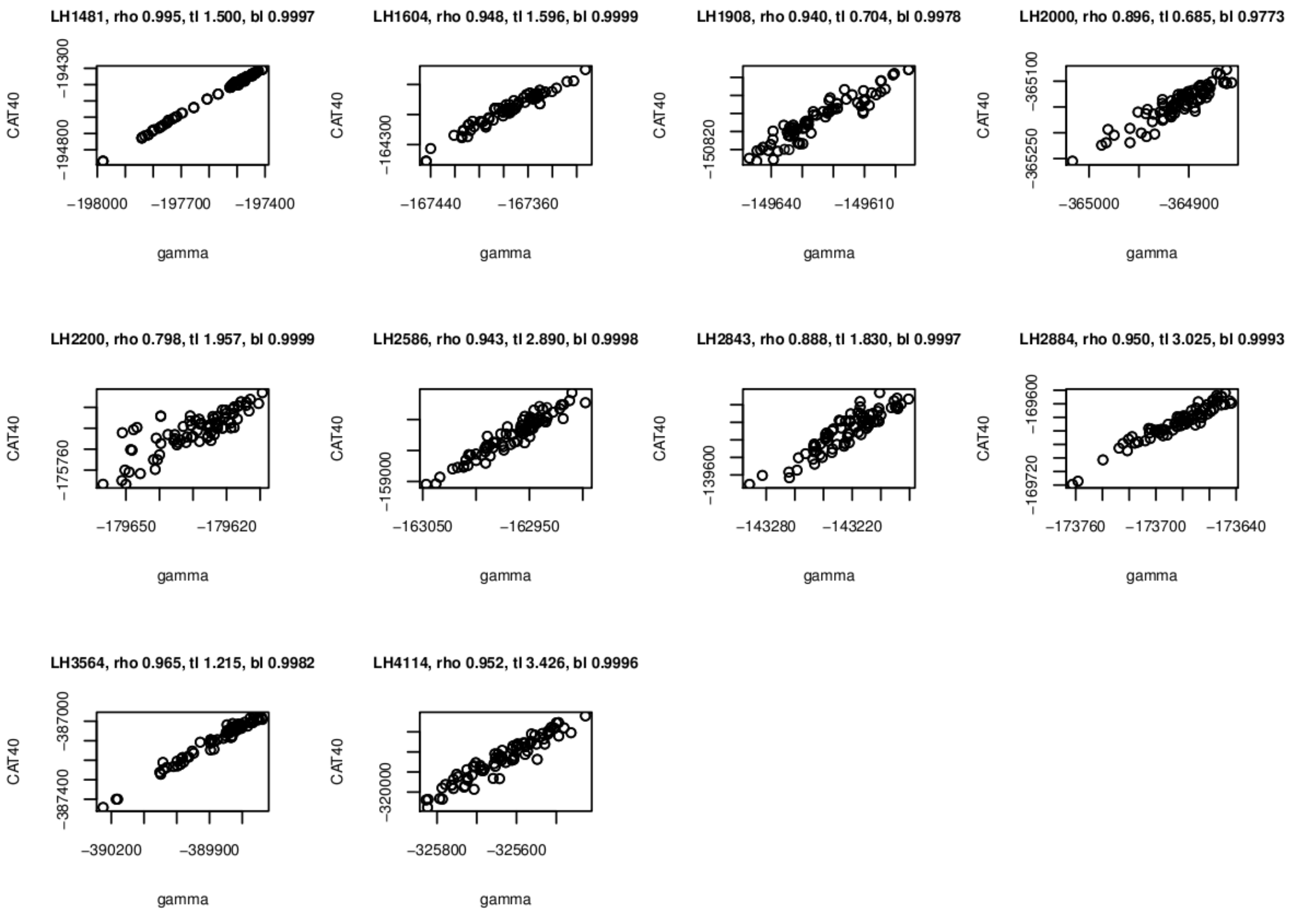

Correlations using the CAT model for the 38k dataset

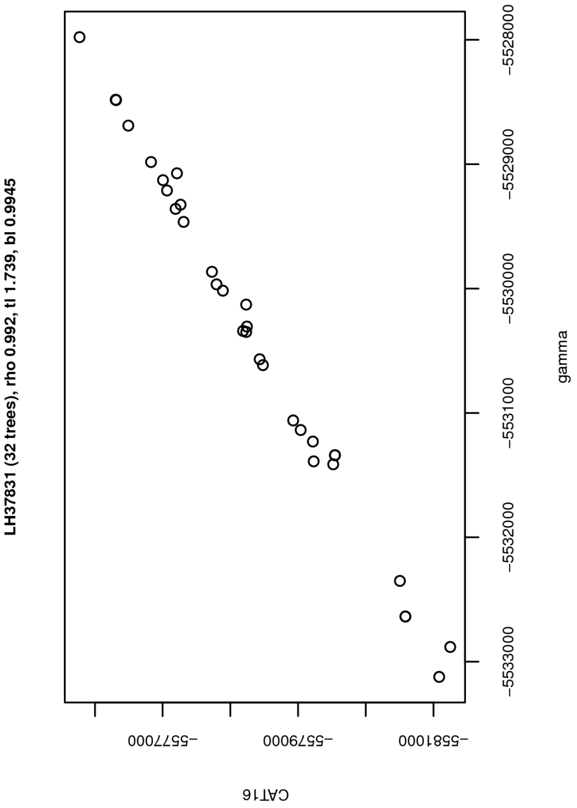

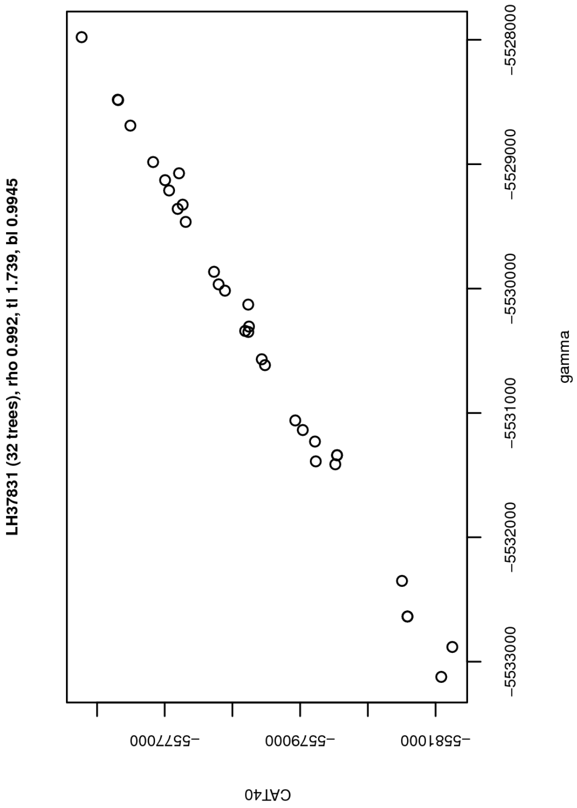

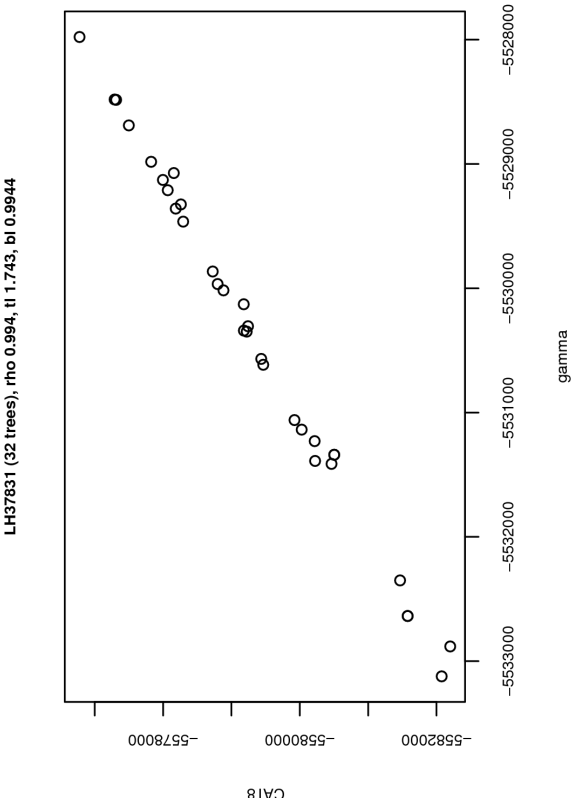

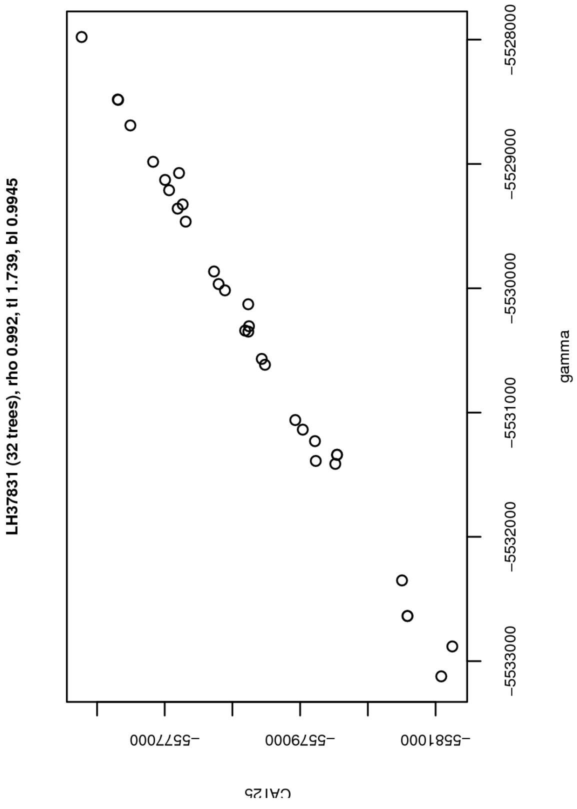

Correlations using the CAT model for the 56k dataset

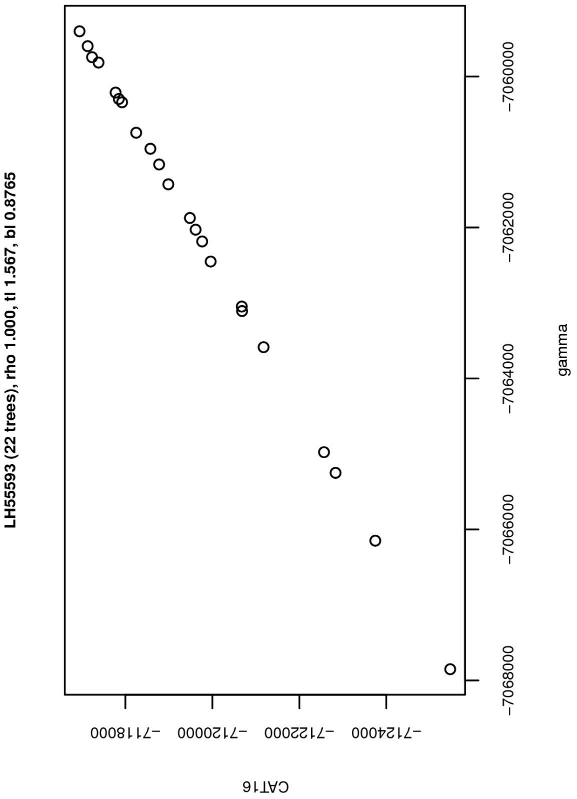

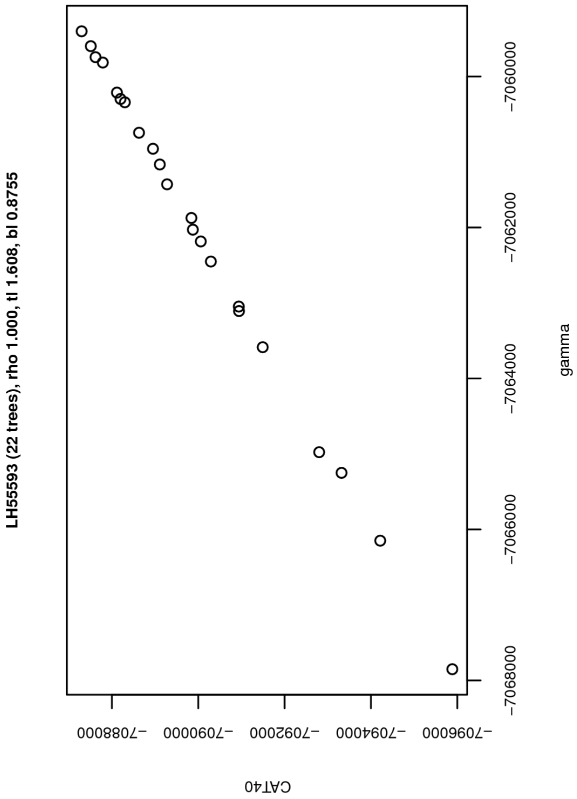

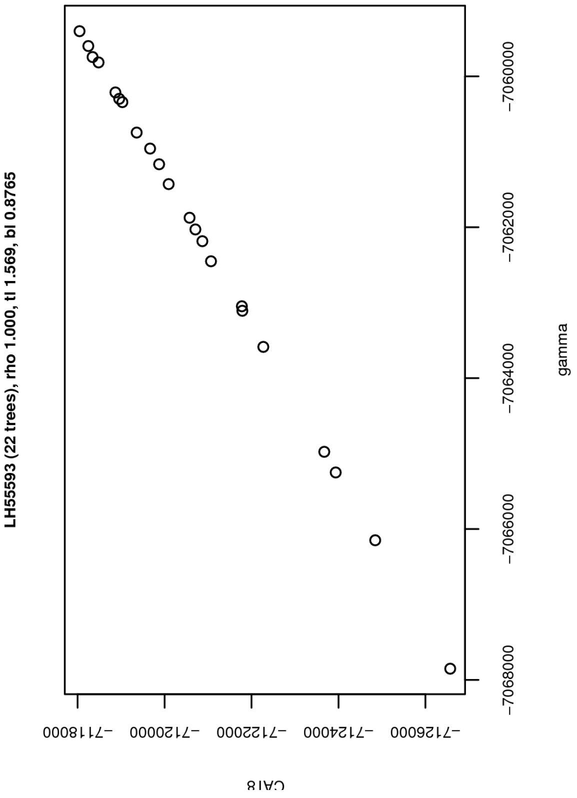

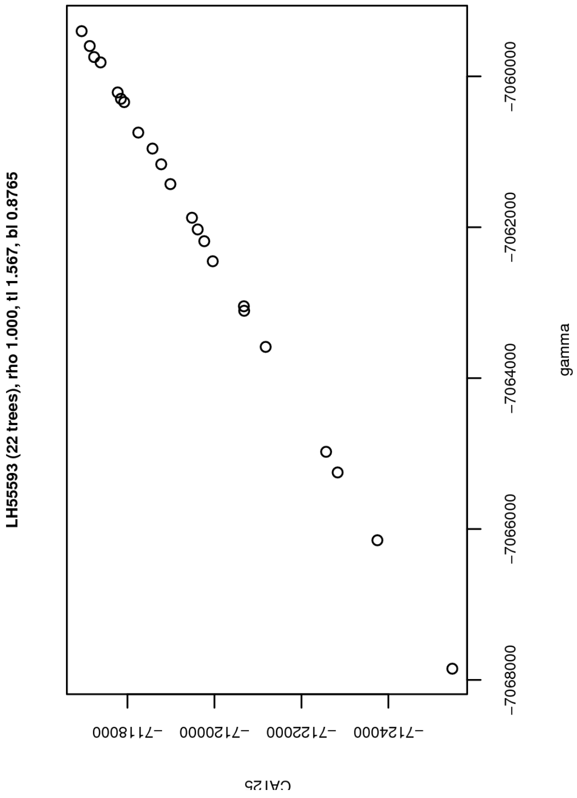

Correlations using the FastTree 2 CAT/Gamma20 model for the 38k and 56k dataset

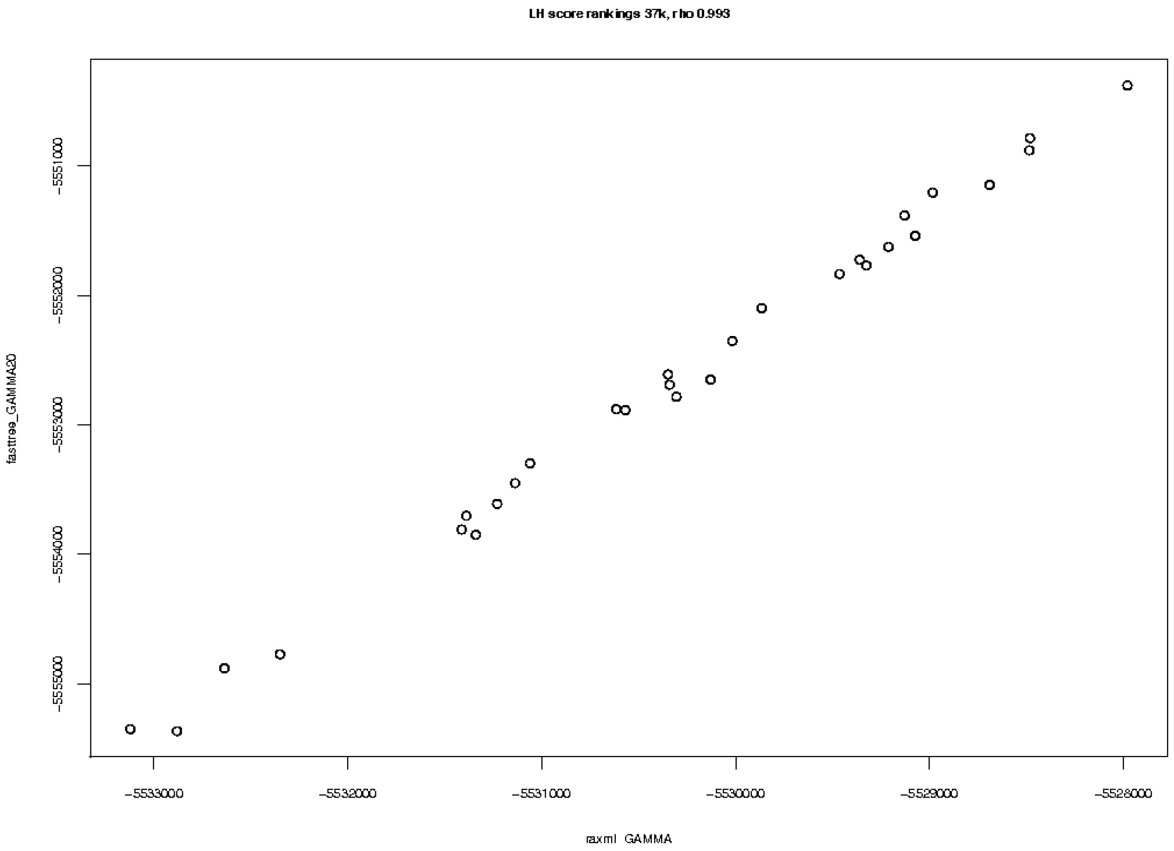

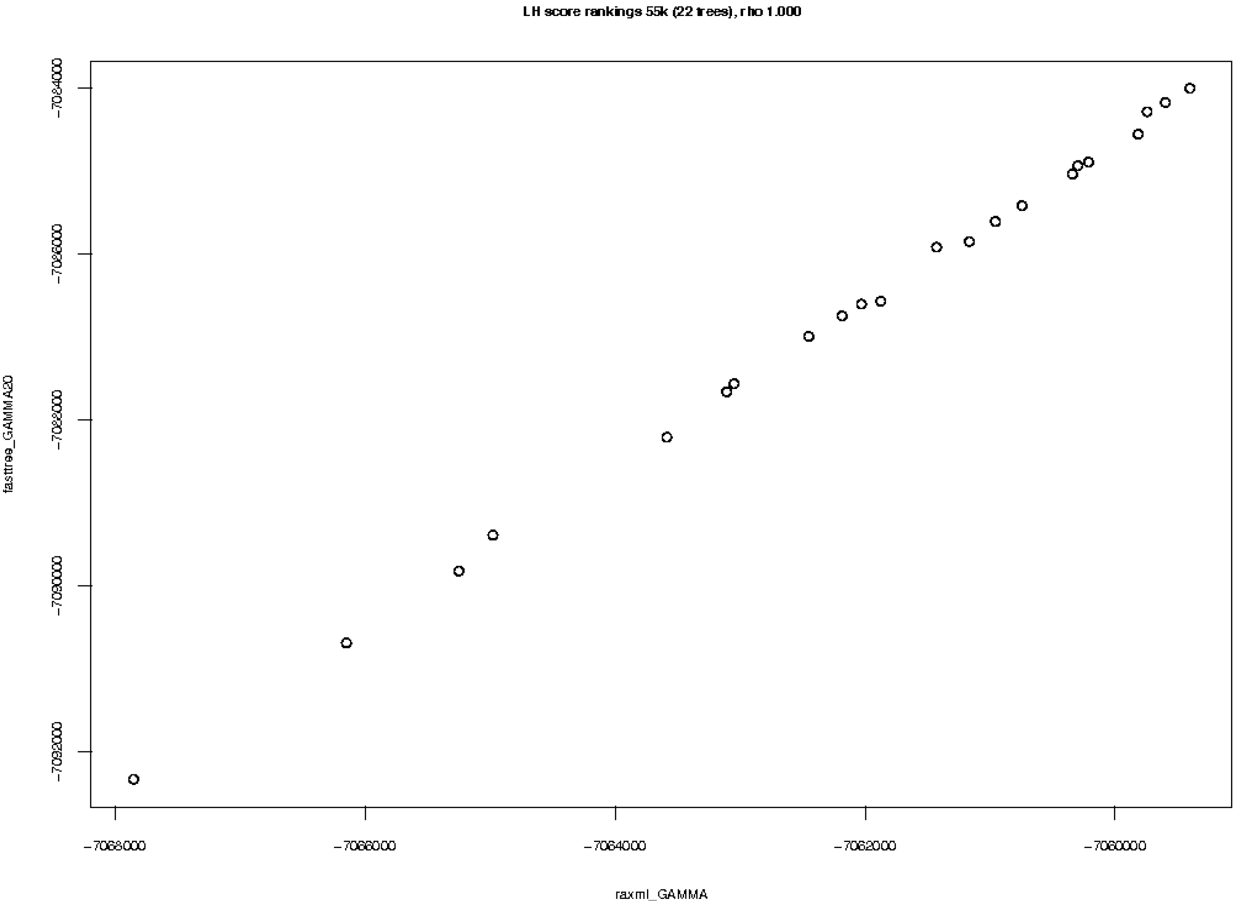

Supplement: Additional file 1 — Supplementary Material. Assesment of alternative criteria to identify the innermost node of a tree. Evaluation of the backbone algorithm with simulated data: Simulation details and symmetric difference for the 5000 taxa dataset, log likelihood scores for ML trees on simulated datasets(1500 and 5000 species). Evaluation of the backbone algorithm with real data and comparison with FastTree 2. Correlation between CAT and Γ-based ML branch length estimates, total tree length ratios, and Spearman rank correlation coefficients between likelihood-induced tree rankings obtained from CAT and Γ for 12 different datasets ranging from 1481 up to 4114 number of taxa. Correlations between log likelihood scores under the RAxML CAT model and Γ model for the 38 k and 56 k dataset. Correlations between log likelihood scores under the RAxML CAT model and the FastTree 2 CAT/Gamma20 model for the 38 k and 56 k dataset. [file 1471-2105-12-470-S1.PDF]
